# Keep Up the Good Work! Age-Moderated Mediation Model on Intention to Retire

**DOI:** 10.3389/fpsyg.2017.01717

**Published:** 2017-10-17

**Authors:** Paola Dordoni, Beatrice Van der Heijden, Pascale Peters, Sascha Kraus-Hoogeveen, Piergiorgio Argentero

**Affiliations:** ^1^Department of Brain and Behavioural Sciences, University of Pavia, Pavia, Italy; ^2^Faculty of Management Sciences, Institute for Management Research, Business Administration, Radboud University Nijmegen, Nijmegen, Netherlands; ^3^Faculty of Management, Science and Technology, Open University of the Netherlands, Heerlen, Netherlands; ^4^Kingston Business School, Kingston University, London, United Kingdom; ^5^Faculty of Economics and Management, HAN University of Applied Sciences, Nijmegen, Netherlands

**Keywords:** older workers, age stereotypes, job support for learning, employability enhancing activities, intention to retire, age-moderated mediation

## Abstract

In European nations, the aging of the workforce is a major issue which is increasingly addressed both in national and organizational policies in order to sustain older workers' employability and to encourage longer working lives. Particularly older workers' employability can be viewed an important issue as this has the potential to motivate them for their work and change their intention to retire. Based on lifespan development theories and Van der Heijden's ‘employability enhancement model’, this paper develops and tests an age-moderated mediation model (which refers to the processes that we want to test in this model), linking older workers' (55 years old and over) perceptions of job support for learning (job-related factor) and perceptions of negative age stereotypes on productivity (organizational factor), on the one hand, and their intention to retire, on the other hand, via their participation in employability enhancing activities, being the mediator in our model. A total of 2,082 workers aged 55 years and above were included in the analyses. Results revealed that the two proposed relationships between the predictors and intention to retire were mediated by participation in employability enhancing activities, reflecting two mechanisms through which work context affects intention to retire (namely ‘a gain spiral and a loss spiral’). Multi-Group SEM analyses, distinguishing between two age groups (55–60 and 61–65 years old), revealed different paths for the two distinguished groups of older workers. Employability mediated the relationship between perceptions of job support for learning and intention to retire in both age groups, whereas it only mediated the relationship between perceptions of negative age stereotypes and intention to retire in the 55–60 group. From our empirical study, we may conclude that employability is an important factor in the light of older workers' intention to retire. In order to motivate this category of workers to participate in employability enhancing activities and to work longer, negative age stereotypes need to be combated. In addition, creating job support for learning over the lifespan is also an important HR practice to be implemented in nowadays' working life.

## Introduction

Defined as “*the continuous fulfilling, acquiring or creating of work through the optimal use of competences*” (Van der Heijde and Van der Heijden, 2006, p. 453), employability has become increasingly important to cope with fast-changing job requirements and to find career opportunities in internal or external labor markets more easily. In view of the aging world population, this also holds true for the category of workers being over 55 years old (cf. European Council, 2001) who have to continue working longer in order to make retirement systems more sustainable (Verbrugghe et al., 2016; Davies et al., 2017). Therefore, in the current scholarly debate, employability has been considered as a personal resource (cf. De Coen et al., 2015) that may influence workers' retirement decisions (Wang and Shultz, 2010; Feldman and Beehr, 2011).

Up until now, however, many employers tend to invest less in the training and development of their older workers compared to their younger counterparts, as they consider investments in them not to pay-off, just because older workers are closer to retirement. Older workers, in turn, may perceive a lack of support for learning in their job (i.e., the degree to which the job design promotes continuous learning and stimulates workers to acquire new knowledge and skills; Tracey and Tews, 2005) and may encounter negative age stereotypes (e.g., regarding older workers being less productive than younger ones; Posthuma and Campion, 2009) which may influence older workers' own expectations regarding the so-called ‘return on investment’ of their participation in HR-activities that are geared at enhancing their employability as well, and, in turn, their decision to stay or leave the labor market (cf. Greller and Simpson, 1999; Schermuly et al., 2014). More specifically, one of the most common age stereotypes regarding older workers is their alleged lower productivity and job performance (Posthuma and Campion, 2009). As a result, companies prefer to seek and hire younger workers and to invest in their further career growth. Furthermore, older workers may feel stigmatized and stereotyped and may opt, therefore, for (earlier) retirement (Schermuly et al., 2014). In other words, a *self-fulfilling prophecy* (Van der Heijden, 2005) may be generated in case older workers don't perceive their job and their organization to stimulate their ongoing personal development, and, as a result, they may even decide to seek new job opportunities elsewhere or to retire (prematurely) (cf. Armstrong-Stassen and Ursel, 2009; Zaniboni et al., 2010; De Wind et al., 2017).

It is well-known that the number of older workers has grown over the past decades, and that the more experience workers accumulate over the years, the more their needs vary in terms of the role work plays in their lives, leading to an ever-increasing workforce heterogeneity (Carstensen, 2006). Consequently, the moderating effect of age is a highly important, yet complex, issue to take into account in scholarly work in this field (cf. Rudolph, 2016; Van der Heijden et al., 2016). Up until now, only few empirical studies have looked into the association between, in particular, older workers' employability and their intention to retire (De Coen et al., 2015; Hennekam, 2015). Moreover, it can be questioned, whether the mechanisms or processes underlying the alleged relationships between job support for learning and negative age stereotypes (being the predictors), employability (being the mediator), and intention to retire (being the outcome) apply to different categories of older workers in similar ways. The present study aims to contribute to the scholarly debate on older workers' employability and their intention to retire (Van der Heijden et al., 2009; De Graaf et al., 2011) by studying the *mediating* role of older workers' participation in employability enhancing activities in the relationships between one organizational (‘perceptions of job support for learning’) and one job-related factor (‘perceived negative age stereotypes on productivity’—from now on called ‘perceived negative age stereotypes’) on their intention to retire, and the *moderating* role of age category in this regard. The following research questions will be addressed: Do older workers' perceptions of job support for learning' and of ‘negative age stereotypes’ in their organization influence their ‘intention to retire’? Are these relationships mediated by older workers' ‘participation in employability enhancing activities’? And are these relationships similar across workers in the age group ranging from 55 to 60 years old compared to those aged from 61 till 65 years old?

This study addresses some relevant issues. First, Van der Heijden's (2005) employability enhancement model hypothesized personal factors, job-related factors and organizational factors to be important determinants of workers' employability, which, in turn, predicts work and career-related outcomes (cf. Van der Heijden et al., 2009; De Cuyper et al., 2011; De Vos et al., 2011; De Coen et al., 2015). The integrative framework to employability enhancement as conceptualized in Van der Heijden's conceptual model builds upon insights from employability research, human resource management and work, and organizational psychology (see Van der Heijden, 2005) and is used as our theoretical basis.

More specifically, much in line with the plea for an integrative approach (De Vos et al., 2011), our theoretical lens will build on this model by looking into age (being an individual-based factor) (comparing employees of 55–60 vs. 61–65 years old), job support for learning (Tracey and Tews, 2005) (as a job-related factor), and perceived negative age stereotypes (Hassell and Perrewe, 1995) (as an organizational factor) in order to predict both workers' employability and intention to retire (being a career-related outcome). We will combine Van der Heijden's (2005) model with insights from the Job Demands-Resources (JD-R) framework (Bakker and Demerouti, 2007), the Conservation of Resources (COR) theory (Hobfoll, 1989), and the retirement literature (e.g., Resources-Based Dynamic Model for Retirement Adjustment; Wang and Shi, 2014).

Second, we will focus on workers in their later career stages (55 and over) in Italy, being one of the countries in Europe with the oldest (working) population (cf. Eurostat, 2015). Because of the serious lack of studies including workers in their late career (cf. De Lange et al., 2010; Müller et al., 2015) and the absence of research on different age groups *within the older workers category*, we will draw upon lifespan developmental theories (Baltes and Baltes, 1990; Carstensen et al., 1999) to investigate the moderating effect of age in the hypothesized relationships.

### Toward a moderated mediation model explaining older workers' intention to retire

#### Perceived job support for learning, perceived negative age stereotypes, and older workers' intention to retire

The design of jobs held by older workers plays an important role in their retirement decisions (cf. Wang et al., 2009) as learning opportunities provided by the job and room for improvement in one's own career can motivate workers to postpone their retirement age (Raemdonck et al., 2015). In line with related studies (Armstrong-Stassen and Ursel, 2009; Schreurs et al., 2011; Hofstetter and Cohen, 2014), we argue that older workers' perceptions of job support for learning (viewed as a job-related resource; cf. Demerouti et al., 2001) may reduce their intention to retire.

In a similar vein, perceived age stereotypes can be seen as a potential antecedent of workers' behaviors (cf. Dordoni and Argentero, 2015), with retirement decisions being no exception (Von Hippel et al., 2013; Schermuly et al., 2014). Specifically in situations where negative stereotypical views on older workers [viewed as a stressful job demand (cf. Demerouti et al., 2001) that require sustained psychological (cognitive and emotional) efforts and that are associated with certain psychological costs (cf. Bakker and Demerouti, 2007)] are prevalent, poorer productivity and job performance are more often reported (Posthuma and Campion, 2009). That is, older workers who feel stigmatized and who are viewed as being less productive may perceive a lack of resources, and may cope with this situation by living up to these negative views and by preparing mentally for earlier retirement (Müller et al., 2013). This may lead to a *self-fulfilling prophecy* (Van der Heijden, 2005) being reflected in reduced investment in further career development by older workers themselves as well, thereby confirming the negative attitude of their supervisors. Based on the theoretical outline presented above, the following was hypothesized:

**Hypothesis 1:** *Older workers' perceptions of job support for learning is negatively related to their intention to retire (H1a), while their perceptions of negative age stereotypes is positively related to their intention to retire (H1b)*.

#### The mediating role of participation in employability enhancing activities

Perceived job support for learning (viewed as a job-related resource cf. Demerouti et al., 2001) can motivate workers to invest in their employability enhancing activities (Pulakos et al., 2000), which refer to those activities that workers undertake to improve and maintain their employability (Van Dam, 2004). In turn, in line with COR theory (Hobfoll, 1989; Wang and Shi, 2014), a so-called ‘gain spiral’ mechanism is argued to enhance workers' employability (viewed as a personal resource; De Cuyper et al., 2012), and, in turn, to reduce their intention to retire (cf. Armstrong-Stassen and Schlosser, 2008; Armstrong-Stassen and Ursel, 2009). In COR theory, resources are defined as “*those objects, personal characteristics, conditions, or energies that are valued by the individual or that serve as a means for attainment of these objects, personal characteristics, conditions, or energies*” (Hobfoll, 1989, p. 516). Examples of resources are mastery, self-esteem, socioeconomic status and employment (Hobfoll, 1989).

Conversely, older workers' perceptions of negative age stereotypes in their organization, viewed as a stressful job demand, can reduce older workers' motivation to participate in employability enhancing activities (Raemdonck et al., 2015). In turn, in line with COR theory (Hobfoll, 1989; Wang and Shi, 2014), a so-called ‘loss spiral’ mechanism is posited to reduce older workers' employability (viewed as a personal resource; De Cuyper et al., 2012), and, in turn, to increase their intention to retire (cf. Armstrong-Stassen and Schlosser, 2008; Armstrong-Stassen and Ursel, 2009). This is in line with Bal et al. (2011) who found that the way workers believe that others perceive them may affect self-based behavioral outcomes at work, such as workers' intentions to retire.

Summarizing, older workers who perceive sustainable support for further career development (Van der Heijden and De Vos, 2015) and who believe that others ‘have faith’ in their potential will be more willing to accumulate resources (cf. Hobfoll, 1989) through participating in employability enhancing activities (De Coen et al., 2015). Subsequently, enhanced employability can motivate workers to postpone retirement (De Coen et al., 2015). Therefore, two processes or mechanisms (reflecting a ‘gain spiral’ and a ‘loss spiral’) can be hypothesized to operate between the work context and older workers' intention to retire. Based on this outline, the following hypothesis was formulated:

**Hypothesis 2**: *Workers' participation in employability enhancing activities (partially) mediates the negative relationship between perceived job support for learning (H2a; reflecting a gain spiral) and the positive relationship between perceived negative age stereotypes (H2b; reflecting a loss spiral), on the one hand, and older workers' intention to retire, on the other hand*.

#### The moderating role of age

According to Selective Optimization with Compensation Theory (SOC) (Baltes et al., 1999), due to the loss of biological, mental, and social resources, older workers are assumed to employ and optimize available resources to reach those goals in life that they consider most desirable and important. This line of reasoning is also followed in research on the so-called ‘prevention focus’ (cf. Higgins, 1998) according to which people strive to minimize or prevent losses of available resources. A prevention focus becomes more salient with age because of the loss of these valuable resources across the lifespan, and even more so if workers perceive a negative ‘loss spiral’. Previous research has already shown a negative relationship between age and growth motives which refers to the value that workers attach to opportunities for advancement and continuous learning (Kooij et al., 2011), as well as between age and learning self-efficacy and learning value (Kochoian et al., 2017). Therefore, workers aged between 55 and 60 years old may benefit more from job support for learning in comparison with workers aged 61–65 years old, as their learning orientation may be relatively stronger (Kanfer and Ackerman, 2004; cf. Kooij and Zacher, 2016; Moghimi et al., 2016). Hence, the ‘gain spiral’ described earlier (Hobfoll, 1989), which may be generated by offering older workers job support for learning, may especially enhance the motivation of those older workers who feel that they have personal resources and a longer ‘time horizon’ (Carstensen, 1995) in order to reach these work-related goals that are salient to them. The concept of time horizon is derived from the Socio-Emotional Selectivity Theory (SST) (Carstensen, 1995) and relates to the narrowing time horizon that workers experience when they grow older over the life cycle. Older workers' perceptions of a more limited time horizon and having fewer (time and energy) resources left is inextricably linked to goal selection and goal pursuit (Carstensen, 2006). Essentially, older workers are likely to adapt to aging by prioritizing emotional regulation as a key life goal, both in general and in their working life in particular (Rudolph, 2016). In fact, based on their limited time horizon, retirement could be an emotionally meaningful goal, particularly so for the oldest workers (Freund, 2008; Henry et al., 2017).

Moreover, the ‘loss spiral’ described earlier (Hobfoll, 1989), which may be generated by older workers perceiving negative age-related stereotyping, may also especially reduce the motivation of those older workers who feel that they have ample personal resources and a longer ‘time horizon’ (Carstensen, 1995) in order to reach work-related goals that are salient to them. In a similar vein, it may be assumed that especially employees aged 61–65 years old strive to maximize their life satisfaction and minimize them being exposed to emotional risks, such as perceived negative age stereotypes. It is conceivable that these older workers will try to avoid exposure to negative events, such as negative age stereotypes, once encountered will try to neglect or ignore them (Rudolph, 2016) and will rather focus on pleasant goals, such as retirement. Those employees who have a relatively more ‘open-ended’ future (which is more applicable to those workers aged 55–60 years old), however, may more easily perceive themselves as being part of their organizations' future workforce. Possibly, negative age stereotypes within the organization may affect employees from the 55–60 years old age category relatively more strongly in comparison with the older age group. Based on the outline provided above, the following two hypotheses were formulated:

**Hypothesis 3:** *Age moderates the strength of the (partially) mediated negative relationship between perceived job support for learning and intention to retire, through participation in employability enhancing activities, such that the (partially) mediated relationship will be stronger for those workers aged 55–60 years old*.
**Hypothesis 4**: *Age moderates the strength of the (partially) mediated positive relationship between older workers' perceptions of negative age stereotypes and intention to retire, through participation in employability enhancing activities, such that the (partially) mediated relationship will be stronger for those aged 55–60 years old*.

## Materials and methods

### Sample and procedure

A survey was conducted in a large Italian financial institution—one of the country's largest banks—with locations in multiple regions. Two thousand seven hundred and eighty five workers aged 55 years and over were invited to participate anonymously in an online intranet survey. Workers were proactively informed about the survey. In addition, two reminders were sent via e-mail to every employee after 2 weeks and after 1 month, respectively. The researchers sampled explicitly across multiple Italian locations of the bank and across different regions. A total of 2,082 respondents (response rate of 74.7%) were included in the final analyses. The majority of respondents (see Table 1) were male (77%), aged between 55 and 60 years old (87%), and held a high school leaving certificate (81%). Moreover, the majority had worked for the bank for more than 30 years (91%). More than half of the respondents had a managerial role (64%). Most of the respondents were married (or cohabiting) (85%), and more than half of them (61%) had at least one child.

**Table 1:** Socio-demographic characteristic of the sample (*N* = 2,082).

**Variable**	***N***	**%**
**GENDER**		
Male	1,670	77
Female	518	23
**AGE**		
55–60 years old	1,921	87
61–65 years old	269	13
**EDUCATION**		
High school leaving certificate	1,729	81
Bachelor/Masters' degree	413	19
**TENURE**		
Less than 30 years old	213	9
More than 30 years old	1,971	91
**JOB LEVEL**		
Office worker	796	36
Manager	1,375	64
**MARITAL STATUS**		
Single/widowed	325	15
Married/cohabiting	1,846	85
**HAVING CHILDREN**		
No	859	39
At least one child	1,309	61

### Measures

All variables were measured using five-point Likert scales (1 = strongly disagree to 5 = strongly agree), were operationalized using thoroughly validated scales, (based on previous English versions), which were carefully derived from the translation-back translation procedure as proposed by Hambleton (1994).

#### Perceived job support for learning

Older workers' perceptions of job support for learning was measured with three items selected from Tracey and Tews (2005) original five-item measurement: “Work assignments include opportunities to learn new techniques and procedures for improving performance”; “There is a strong belief that continuous learning is important to successful job performance”; and “Gaining new information about ways to perform work more effectively is important in this organization” (Cronbach's alpha was 0.79).

#### Perceived negative age stereotypes

Older workers' perceptions of negative age stereotypes regarding their lower productivity in the organization was measured with four items selected from Henkens (2005). The four selected items referred to job performance and autonomy: “In my workplace people think that older workers are less productive than younger workers”; “In my workplace people think that older workers are less creative than younger workers”; “Older workers are just as enterprising as younger workers”; and “Older workers prefer not to be assigned tasks by younger workers” (Cronbach's alpha was 0.66).

#### Participation in employability enhancing activities

Older workers' participation in employability enhancing activities was measured with three items selected from Van Dam's (2004) original five-item measurement scale. The items referred to the development of knowledge and career enhancement: “I am actively trying to develop my knowledge and work experiences”; “I do a lot to manage my career”; and “I am actively trying to increase my employability” (Cronbach's alpha was 0.71).

#### Intention to retire

Following Zaniboni et al. (2010), older workers' intention to retire was measured using three items: “Even when I can retire I will keep on working”; “I will keep on working by changing job type, even when I can already retire”; and “As soon as I can retire, I will definitely stop working” (Cronbach's alpha was 0.66).

#### Moderator

The moderating variable “age” was measured by means of a dummy variable representing two age categories: (1) workers aged 55–60 years old; and (2) those aged 61–65 years old.

#### Control variables

Given the outcomes of previous empirical studies, gender, education, tenure, job level (De Coen et al., 2015), marital status (Elovainio et al., 2005), and having children (Oakman and Wells, 2013) were included as control variables in the analyses. The following scales were used: gender (0 = female, 1 = male), education (0 = high school, 1 = university), tenure (0 = less than 30 years employed at the bank, 1 = more than 30 years employed at the bank), job level (0 = office worker, 1 = manager), marital status (0 = single/widowed, 1 = married/cohabiting), and having children (0 = no, 1 = at least one child).

### Analyses

Mplus (Muthèn and Muthèn, 2012) was used to perform the analyses. First, the divergent validity of our latent constructs was examined by testing the measurement model using Confirmatory Factor Analysis (CFA). Second, Structural Equation Modeling (SEM) was used to test our hypotheses. The fit of the hypothesized model was compared to that of several competing models, using the full sample. Subsequently, the best fitting model was selected to examine the invariance of the model across the two distinguished employee age groups. Third, structural invariance was tested using Multi-Group SEM analyses. The data was split into two groups based on the dummy variable for age. The first group included respondents in the age range from 55 to 60 years old (*N* = 1,815). The second group included those respondents aged 61–65 years old (*N* = 267).

## Results

### Preliminary analyses

The correlation matrix (see Table 2) shows that perceived job support for learning was positively associated with participation in employability enhancing activities, while it was negatively associated with perceived negative age stereotypes, and with workers' intention to retire. Table 2 also shows a negative association between perceived negative age stereotypes and participation in employability enhancing activities, and a positive association with intention to retire. Based on this, the mediation paths from perceived job support for learning and perceived negative age stereotypes on intention to retire, through participation in employability enhancing activities, are plausible and, therefore, further investigation of these using SEM analyses can be justified (Baron and Kenny, 1986).

**Table 2:** Correlation matrix (*N* = 2,082).

**Variable**	**1**	**2**	**3**	**4**	**5**	**6**	**7**	**8**	**9**	**10**
1. Perceived Job support for learning	–									
2. Perceived negative age stereotypes	−0.23^***^	–								
3. Participation in employability enhancing activities	0.56^***^	−0.22^***^	–							
4. Intention to retire	−0.32^***^	0.16^***^	−0.47^***^	–						
5. Gender	0.00	−0.012	−0.03	−0.14^***^	–					
6. Education	−0.01	0.05	0.12^***^	−0.13^***^	0.01	–				
7. Tenure	−0.09^*^	0.07	−0.14^**^	0.11^**^	0.06	−0.51^***^	–			
8. Job level	0.18^***^	−0.04	0.32^***^	−0.30^***^	0.42^***^	0.27^***^	0.14^**^	–		
9. Marital status	0.09^*^	−0.02	0.06	−0.11^**^	0.32^***^	−0.04	0.06	0.14^***^	–	
10. Having children	0.04	0.06	0.05	−0.19^***^	0.21^***^	0.13^***^	−0.17^***^	0.10^**^	0.47^***^	–

*** p < 0.001;

** p < 0.01;

* *p < 0.05*.

### Testing the measurement model

A full measurement model was tested including the latent constructs of: (1) perceived job support for learning; (2) perceived negative age stereotypes; (3) participation in employability enhancing activities; and (4) intention to retire. Using CFA, the four latent variables constructed by means of 13 observed variables were modeled. The full measurement model yielded a good fit to the data (see Table 3, Model C). All observed variables appeared to have a significant contribution to the latent constructs with factor loadings for the items concerned ranging from 0.90 to 0.29. Therefore, all the manifest variables were included in comprising the latent constructs (Anderson and Gerbing, 1988; Iacobucci, 2009).

**Table 3:** Goodness of fit indices for the distinguished models (*N* = 2,082).

		**Chi-square test of model fit**	**DF**	**Model comparison (Δ Chi-square)**	**Δ DF**	**RMSEA**	**CFI**	**TLI**	**SRMR**
A	(M ->DV)	728.378^***^	145			0.044	0.931	0.920	0.047
B	(IV-> DV)	829.245^***^	144	−100.867^***^ (A–B)	1	0.048	0.919	0.905	0.053
C	(IV -> M -> DV)	718.757^***^	143	110.488^***^ (B–C)	1	0.044	0.932	0.920	0.045
				9.621^**^ (A–C)	2				

*M, mediator; DV, dependent variable; IV, independent variable*.

*** p < 0.001.

** *p < 0.01*.

### Testing the hypothesized model

The fit of the hypothesized mediation model was compared with that of several competing models, using the full sample. Afterwards, the best fitting model was used to examine the invariance of the model across the two distinguished age groups. As Table 3 shows, the fit of Model A [including the direct relationship between the mediator (participation in employability enhancing activities) and the outcome (intention to retire)] was acceptable. In line with our hypotheses, participation in employability enhancing activities was negatively associated with intention to retire (β = −0.38; *p* < 0.001). Next, the fit for Model B was computed, in which direct effects from the independent variables (perceived job support for learning and perceived negative age stereotypes) on the dependent variable (intention to retire) were tested. The fit indices suggested that the model fit was acceptable (see Table 3). In support of Hypothesis 1, perceived job support for learning (β = −0.24; *p* < 0.001) was negatively related to intention to retire (H1a), while perceived negative age stereotypes (β = 0.06; *p* = 0.017) related positively to intention to retire (H1b). Subsequently, the mediation model (Model C) was calculated, which included direct and indirect paths from perceived job support for learning and perceived negative age stereotypes to intention to retire, through participation in employability enhancing activities. In line with Hypothesis 2a, the partial mediation of perceived job support for learning on intention to retire, through employability enhancing activities (β = −0.16; *p* < 0.001) was confirmed, also leaving a direct effect of job support for learning on intention to retire (β = −0.07; *p* = 0.027). Moreover, in line with Hypothesis 2b, model C confirmed a full mediation of perceived negative age stereotypes on intention to retire, through employability enhancing activities (β = 0.02; *p* = 0.005), whereas the direct effect was no longer significant (β = 0.05; *p* = 0.066). The mediation model provided an acceptable fit to the data. Moreover, the outcomes of the Chi-square change indicated that the fit of the mediation model was superior to the fit of the direct effects' models (Models A and B) (see Table 3). Therefore, the mediation model was used for testing the moderation hypotheses.

### Testing for measurement invariance

In order to investigate the possible moderation effects of employee age, by means of Multi-Group SEM analyses, the latent constructs first needed to be checked for measurement invariance among the groups. Following the procedure by Van de Schoot et al. (2012), the measurement invariance of the mediated model (Model C) was studied across the two distinguished age groups. Three models were estimated and compared regarding their model fit. The fit of the model with unconstrained parameters, the so-called configurational model (Model 1), was compared with the model in which the factor loadings were constrained to be equal, the so-called ‘metric model’ (Model 2), and with the model in which both factor loadings and intercepts were constrained to be equal, the so-called ‘scalar model’ (Model 3). The results of the model fit tests (see Table 4) showed a significantly worse fit of the ‘configurational model’ (unconstrained) in comparison to both the metric (factor loadings constrained) and ‘scalar model’ (factor loadings and intercepts constrained). The scalar model was preferred over the metric model, as it did not have a significantly worse fit (Van de Schoot et al., 2012). This implies that the meaning of the constructs, based on both the factor loadings and the intercepts, is equal in both age groups. Therefore, it is justified to compare the two age groups regarding their scores on the latent constructs and the model structure (Van de Schoot et al., 2012).

**Table 4:** Measurement fit for the distinguished invariance models (*N* = 2,082).

	**Configural model (1)**	**Metric model (2)**	**Scalar model (3)**	**CHI square difference test (CHI square difference/DF)**
AIC	70631.337	70623.783	70628.285	1–2^***^ (10.446/9)
BIC	71139.034	71080.711	71034.443	1–3^***^ (32.948/18)
CFI	0.956	0.956	0.955	2–3 (22.502/9)
TLI	0.942	0.946	0.948	

*** *p < 0.001*.

### Testing the moderated mediation model for intention to retire

**Figure 1:**
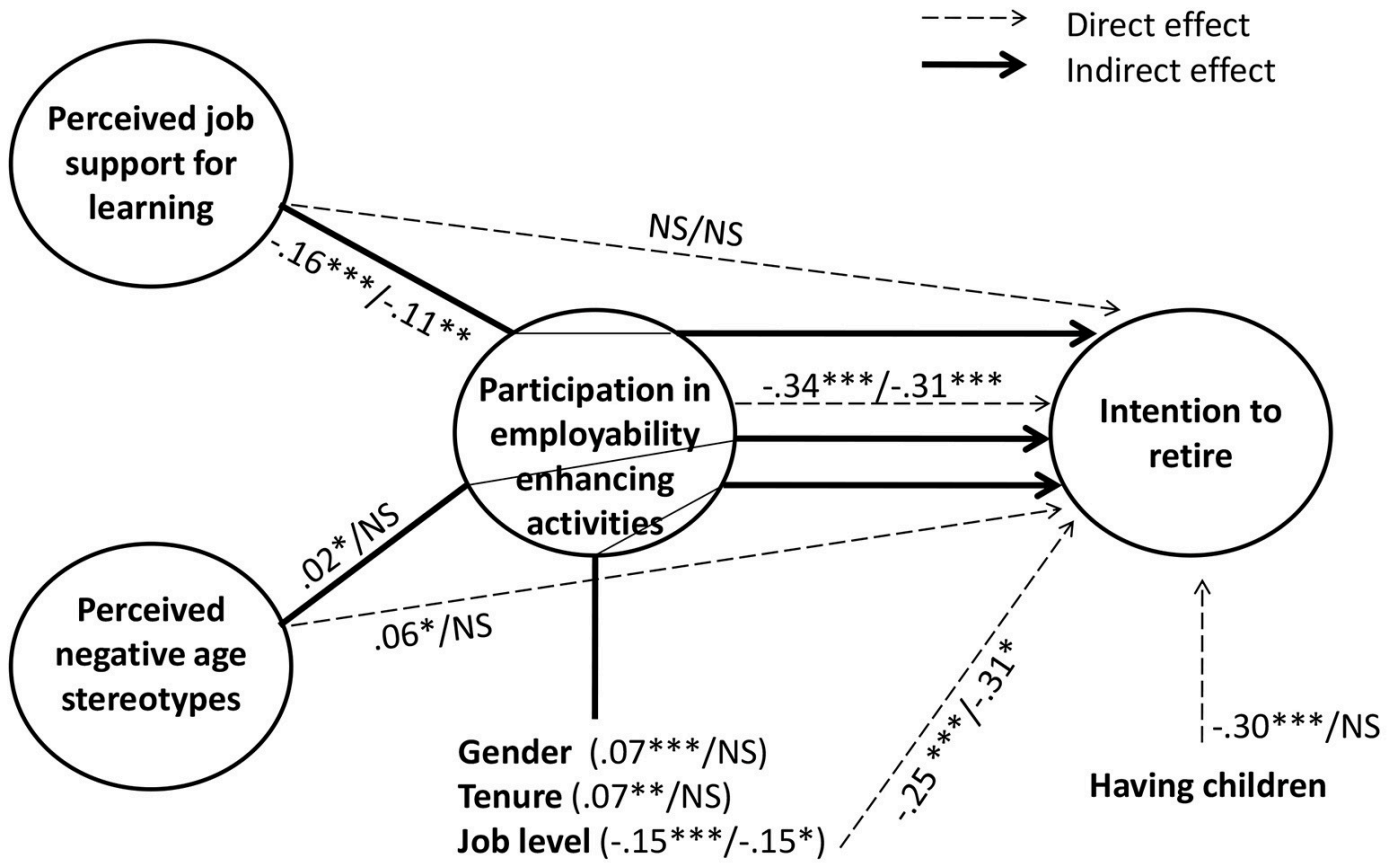
Parameter estimates of the Multi-Group SEM (Standard coefficients). The coefficients of the different employees age groups are sorted in ascending order: 55–60 years old/61–65 years old. ***p < 0.001; **p < 0.01; *p < 0.05.

Hypotheses 3 and 4 stated that employee age would moderate the (partially) mediated relationships between, respectively, perceived job support for learning and perceived negative age stereotypes, on the one hand, and intention to retire, on the other hand. Specifically, we expected that those relations were stronger among those workers aged between 55 and 60 years old. Results of the Multi-Group SEM tests indeed revealed different paths across the two age groups. Table 5 shows the results for each employee age group. Figure 1 presents the model outcomes with standardized path coefficients and significance levels for the age groups of workers between 55 and 60 years old vs. those 61–65 years old.

**Table 5:** Structural path analyses.

**Variables**	**Intention to retire**	
	**55–60 results**	**61–65 results**
	**β (SE)**	**β (SE)**
**DIRECT EFFECTS**		
Perceived job support for learning	−0.06 (0.03)	−0.06 (0.08)
Perceived negative age stereotypes	0.06^*^ (0.03)	−0.01 (0.07)
Participation in employability enhancing activities	−0.34^***^ (0.03)	−0.31^***^ (0.09)
Gender	−0.09 (0.06)	−0.01 (0.27)
Education	−0.11 (0.07)	0.27 (0.17)
Tenure	0.11 (0.09)	0.35 (0.33)
Job level	−0.25^***^ (0.06)	−0.31^*^ (0.16)
Marital status	0.01 (0.07)	−0.24 (0.23)
Having children	−0.30^***^ (0.05)	−0.22 (0.14)
**INDIRECT EFFECTS**		
Perceived job support for learning ON Intention to retire VIA Participation in employability enhancing activities	−0.16^***^ (0.02)	−0.11^**^ (0.037)
Perceived negative age stereotypes ON Intention to retire VIA participation in employability enhancing activities	0.02^*^ (0.01)	
Gender ON Intention to retire VIA Participation in employability enhancing activities	0.07^***^ (0.02)	
Tenure ON Intention to retire VIA Participation in employability enhancing activities	0.07^*^ (0.03)	
Job level ON Intention to retire VIA Participation employability enhancing activities	−0.15^***^ (0.02)	−0.15^*^ (0.06)
R^2^	0.22^***^ (0.02)	0.17^***^ (0.06)

*** p < 0.001;

** p < 0.01;

* *p < 0.05*.

First, in both age groups, perceived job support for learning appeared to be negatively related to intention to retire, via participation in employability enhancing activities. Yet, in line with our expectations, the negative relationship was stronger among the 55–60 years old category (β = −0.16, *p* < 0.001) in comparison with the category of workers 61–65 years old (β = −0.11, *p* = 0.003). Taking together the fact that there is no structural invariance, and that workers 55–60 years old have higher beta coefficients, Hypothesis 3 was supported with our data.

Second, perceived negative age stereotypes related positively to intention to retire, via participation in employability enhancing activities for workers aged 55–60 years old (β = 0.02, *p* = 0.02). However, there was no significant relationship for the workers aged 61–65 years old. Moreover, the relationship between participation in employability enhancing activities and intention to retire was stronger for the workers between 55 and 60 years old in comparison to the workers 61–65 years old. With these outcomes, concerning the (partial) mediation path of perceived negative age stereotypes, Hypothesis 4 was supported by our data as well.

## Discussion and conclusions

The present study aimed to contribute to the debate on older workers' sustainable employability and retirement considerations by thoroughly testing a new empirical model, based on Van der Heijden's (2005) employability enhancement framework, while incorporating additional key scientific literature. It was intended to unravel the relationships between older workers' perceptions of job support for learning and negative age stereotypes, their participation in employability enhancing activities, and their intention to retire. Using our full sample, empirical evidence for two mediation paths, wherein older workers' participation in employability enhancing activities acted as a mediator was found. More specifically, partial mediation was found for the relationship between perceived job support for learning and intention to retire, while full mediation was found for the relationship between perceived negative age stereotypes and intention to retire. Furthermore, empirical support was found for the moderation hypotheses predicting that these mediation effects differed among the two distinguished age groups. In line with our expectations, the mediation effect was stronger for workers aged 55–60 years old in comparison with their relatively older counterparts (61–65 years old). Below, an elaboration of the main results of our study is provided.

In line with related studies (Armstrong-Stassen and Ursel, 2009; Schreurs et al., 2011; Hofstetter and Cohen, 2014), our empirical work revealed that perceived job support for learning [viewed as a job-related resource (cf. Demerouti et al., 2001)] has the potential to reduce older workers' intention to retire. Further, in line with earlier scholarly work (cf. Von Hippel et al., 2013; Hofstetter and Cohen, 2014; Schermuly et al., 2014), it was found that perceived negative age stereotypes [viewed as an organizational demand (cf. Demerouti et al., 2001)], was positively associated with intention to retire. Hence, both the job-related and the organizational factor that we included in our model (i.e., positive perceptions on the room for development in their job, and negative perceptions on their organization's culture, in terms of the prevalence of negative age stereotyping) have proven to be important factors in older workers' retirement intentions (cf. Carr et al., 2016).

Investigating the direct relationship between older workers' participation in employability enhancing activities and intention to retire provided empirical support for the theoretical claim that employability [(viewed as a personal resource; cf. De Cuyper et al., 2012; De Coen et al., 2015)] has the potential to mediate relationships between job-related and organizational characteristics, such as job resources (i.e., job support for learning) and job demands (i.e., perceived negative age stereotypes), on the one hand, and older workers' intention to retire, on the other hand (cf. Bakker et al., 2003). Our path model revealed two important behavioral mechanisms operating between the work context and older workers' intention to retire.

On the one hand, building upon the JD-R model (Bakker and Demerouti (2007), COR theory (Hobfoll, 1989), and the Resources-Based Dynamic Model for Retirement Adjustment (Wang and Shi, 2014), the present research found a solid ‘positive gain spiral’ wherein older workers' perceptions of job support for learning motivates them to participate in employability enhancing activities (conservation of personal resources), which, in turn, reduces their intention to retire (cf. De Cuyper et al., 2011). Possibly, individuals who have accumulated more personal resources, as a result of them having had better chances to grow in their job, feel that their sustainable employability allows them to prolong their working life.

On the other hand, a ‘negative loss spiral’ (Hobfoll, 1989) was found as well, in which older workers' perceptions of negative age stereotypes discourages their participation in employability enhancing activities, which, in turn, increases their intention to retire. Conceivably, older workers' perceptions of negative age stereotypes lower their self-evaluations, which, through a *self-fulfilling prophecy*, reduces their participation in employability enhancing activities (Van der Heijden, 2005). This, in turn, results in them wanting to leave their working organization sooner. With these outcomes, the present study follows Finkelstein et al. (2015) and calls for a broader perspective on age-related stereotyping, including more empirical work on meta stereotyping (i.e., better understanding of cross-age dynamics, and improving interactions in age-diverse work settings). That is to say, the daily pulse of interpersonal interaction in an organization requires attention for multiple parties involved (such as older workers, colleagues, and managers), which altogether have the potential to affect individual behaviors.

Finally, these findings also confirm the outcomes of a recent contribution by Zaniboni (2015) in which older workers who still had access to personal resources (i.e., learning ability and interest in development) desired to go on working longer in case they perceived a low amount of age discrimination.

Finally, the present research contributed to the debate on older workers' sustainable employability (Van der Heijden et al., 2009; De Graaf et al., 2011) by looking into the rather complex moderating effects of age within the studied relationships. Our multi-group SEM findings supported the assumption that there is a relationship between individuals' so-called ‘life time horizon’ (cf. Carstensen, 2006) and participation in employability enhancing activities, implying that those aged 55–60 years old have a more ‘open-ended future’ in comparison with those aged 61–65 years old, and are more inclined to invest in their further career development (cf. Kochoian et al., 2017) when supported by their jobs. Additionally, in line with lifespan developmental theories (Baltes and Baltes, 1990; Carstensen et al., 1999) and empirical evidence from previous aging research (cf. Baltes et al., 1999; Kanfer and Ackerman, 2004; Freund, 2008; Kooij et al., 2011; Kooij and Zacher, 2016; Moghimi et al., 2016; Rudolph, 2016), our multi-group SEM results confirmed that, with aging, people become more motivated toward maintaining the performance in their current job, instead of focusing on growth and development goals (i.e., being less learning-oriented), and tend to minimize losses (i.e., paying less attention to, focusing less on or avoid situations wherein they encounter negative age stereotypes, and as a result, probably, being less threatened by these).

In particular, our multi-group SEM findings supported outcomes from earlier work suggesting a stronger ‘prevention focus’ (Higgins, 1998) among older workers (Ebner et al., 2006), since the allocation of resources by individuals for acting upon growth goals tends to decline throughout the lifespan while, with aging, the allocation of resources for acting upon maintenance and regulation of ‘loss or prevention’ goals appear to increase (see Freund, 2008; Rudolph, 2016). Concrete, older workers may prefer to focus on what they might gain in their work setting rather than what they might lose. In doing so, they may shift their attention from focusing upon a further career growth to a possible alternative, in this case retirement. It is likely that in the meantime they may value their current position, in particular the positive relationships with their colleagues, and may be less bothered and/or threatened by negative age stereotyping. Moreover, in perceiving negative age stereotypes, older workers may re-evaluate the meaning of work (cf. Hobfoll, 1989; Baltes and Baltes, 1990), resulting in a decreased value that work has for them, which, in turn, may increase their likelihood to retire.

The revealed employee age group effects in this study may be explained by highlighting the 55–60 years old age group's relatively ‘open perception of the future’ in comparison with the 61–65 years old age group who will have a relatively ‘limited time perspective’ (Carstensen et al., 1999). That is, the former age group still has (almost) 10 years of work ahead, whereas the latter may be already more disengaged from their organization's prevailing culture. In fact, the relatively younger employee age group may see retirement more as a future event and, therefore, might be more discouraged by negative organizational factors, in our case perceptions of negative age stereotypes, as these may affect (harm) their actual mindset and immediate future. Alternatively, the 61–65 years old group's awareness of reaching retirement might contribute to striving for experiences that are more positive (for instance focusing on good relationships with close colleagues) in order to get rid of these negative emotions (Carstensen et al., 1999; Henry et al., 2017).

Overall, our multi-group results contribute to the existing literature by applying lifespan developmental theories in a study focusing on ‘older workers’ age brackets. In fact, the present research has demonstrated that, due to its heterogeneity, older workers' age categories should not be taken as one unit of analysis. Based on the effects of age that were found in this contribution, it can be concluded that further elaborating on age-related processes incorporating a ‘future time perspective’ is strongly needed in order to advance the HRM, career and employability literatures, since it allows scholars to better understand *why* and *how* age effects occur and how they affect organizational behaviors (cf. De Lange et al., 2010; Rudolph, 2016).

Despite the important contributions of our study as discussed above, there are several limitations that should be acknowledged. First, the data were cross-sectional. Therefore, our conclusions have to be interpreted with care. Second, as all variables in our study were measured using self-reports, a common-method bias may exist (Podsakoff et al., 2003). Moreover, although we believe that the scales reflect meaningful content coverage and uni-dimensionality, implying that the relatively low alphas are not a major impediment to its use (Schmitt, 1996), more reliable measures for perceived negative age stereotypes and for intention to retire might be used in future work. In response to the limitations that were reported above, future longitudinal research, preferably using multi-wave designs in order to better examine and analyze in-depth the causal relationships within the model (De Lange, 2005), and incorporating more objective data, such as, actual retirement age, is called for.

We are aware of the fact that our sample is characterized by a higher percentage of male workers, as in most banking organizations. That is, in future research, both gender and age could be taken into account in multi-group analyses. Moreover, it needs to be said that (in Italy) older workers are more likely to have a managerial role because of their seniority in the organization, being a possible reason for the relatively large amount of respondents who held a managerial position. Therefore, future work is needed in order to rule out possible effects of organizational roles and positions.

Our study has important implications for managers and other key stakeholders in organizations who are in search for measures to enhance older workers' career development, in order to improve both their workers' sustainable employability as well as the performance of their organization. Increasing older workers' added value throughout their lifespan strengthens their chances in both the internal and external labor market (Van der Heijden et al., 1998; Rocco and Thijssen, 2006; Zappalà et al., 2008). In order to achieve these multiple goals, organizations should provide all their workers, with older workers being no exception, with ample job support for learning and should combat a culture that is prone to negative age stereotypes. With regard to the latter, human resource policies should pay more attention to the *process* of aging and should focus on protecting sustainable employability throughout workers' entire career in order for older workers to feel ‘conserved’ instead of ‘depreciated’ (Peterson and Spiker, 2005; Beach, 2016). Moreover, our study shows that older workers respond differently to job-related and organizational factors than younger workers (cf. Zacher et al., 2017). Our results call for a reconsideration of reflections on ‘return on investment’ regarding the oldest workforce that are ubiquitous and solely based on negative age stereotyping rather than on actual strengths and weaknesses of the specific employee involved. Zwick (2011) highlighted that, often, organizations do not offer appropriate learning opportunities to older workers because firms may view older workers as being less effective; that is, on-the-job training is rarely available for the older counterparts of the workforce. This while the present results show that support for learning increases their participation in employability enhancing activities, and, as a result, reduces their intention to retire. Hence, job design models in working organizations should take into account workers' changing needs across the lifespan (see also Henry et al., 2015). Using a lifespan perspective and carefully paying attention to age-related processes (such as workers' changing future time perspective and their prevention focus), human resource specialists and line managers should focus on supporting older workers' development throughout their entire career.

## Ethics statement

This study was carried out in accordance with the recommendations of University of Pavia with written informed consent from all subjects. All subjects gave written informed consent in accordance with the Declaration of Helsinki.

## Author contributions

PD: conceptualization, data curation, formal analysis, investigation, methodology, project administration, visualization, writing original draft, writing review, and editing; BV and PP: conceptualization, formal analysis, investigation, methodology, supervision, visualization, writing original draft, writing review, and editing; SK: data curation, formal analysis, methodology, software, writing review, and editing; PA: data curation, funding acquisition, resources, supervision, writing review, and editing.

### Conflict of interest statement

The authors declare that the research was conducted in the absence of any commercial or financial relationships that could be construed as a potential conflict of interest. The reviewer SC and handling Editor declared their shared affiliation.
